# Whole-genome sequencing of *Salmonella pullorum* and Sanguinarine-induced mutant

**DOI:** 10.1128/MRA.00095-23

**Published:** 2023-08-18

**Authors:** Huahai Chen, Zongyan Li, Aijie Hou, Yeshi Yin

**Affiliations:** 1 Key Laboratory of Comprehensive Utilization of Advantage Plants Resources in Hunan South, Hunan Engineering Research Center for Research and Development of Plant Resources in Nanling Area, College of Chemistry and Bioengineering, Hunan University of Science and Engineering, Yongzhou, Hunan, China; 2 School of Biological Science and Technology, Hunan Agricultural University, Changsha, Hunan, China; University of Maryland School of Medicine, Baltimore, Maryland, USA

**Keywords:** *Salmonella pullorum*, Sanguinarine

## Abstract

This work was performed on commercially purchased *Salmonella pullorum* CVCC519 originally isolated from chicken intestinal content. The Sanguinarine-resistant strain XM3104 was isolated from Sanguinarine-induced CVCC519. To identify possible mechanisms underlying resistance, the complete genomes of CVCC519 and XM3104 were sequenced using PromethION and next-generation sequencing.

## ANNOUNCEMENT

*Salmonellosis* is one of the leading causes of death among poultry worldwide (1), and the growing threat of antibiotic resistance is causing a healthcare crisis. Sanguinarine, a plant-derived feed additive, has become popular in animal breeding (2). To detect resistance to Sanguinarine, CVCC519 was ordered from China Veterinary Culture Collection Center and soaked in Luria Bertani(LB) broth containing 0.125 mg/mL Sanguinarine (minimum inhibitory concentration) at 37℃ for 3 d. The mutant XM3104 was isolated on an LB plate containing Sanguinarine (0. 25 mg/mL).

For genomic DNA extraction, CVCC519 was cultured in LB broth, and XM3104 was cultured in LB broth containing Sanguinarine, both at 37℃ for 3 days. DNA was extracted using the kit of QiagenGenomic-Tip20/G; DNA quality was examined by electrophoresis, Nanodrop spectrophotometers, and Qubit Fluorometric. An amount of 20 kb of DNA fragments was purified using Blue Pippin (Sage Science). Adapter ligation was performed with Native Barcoding Kit (SQK-NBD114.24). The DNA library was quantified using Qubit. Real-time single-molecule sequencing was performed on the Nanopore PromethION sequencer (R9.4.1). In summary, 109,405 reads (mean length 22446.27 bp and reads N50 31,084 bp) and 3,567,142,532 bp raw data were generated for CVCC519, and 173,015 reads (mean length 20617.53 bp and reads N50 29,645 bp) and 2,455,734,303 bp raw data were generated for XM3104. Guppy (version: 5.0.16) was used for Oxford Nanopore Technologies (ONT) basecalling, mean_qscore_template ≥7 and sequence length ≥1,000 bp were used for quality control. After quality control, 2,315,096,934 bps were obtained for CVCC519 and 3,302,783,638 bps for XM3104. Flye Version 2.9 (https://github.com/fenderglass/Flye, parameter: off) was used for assembly (3) and Pilon Version 1.23 (https://github.com/broadinstitute/pilon, parameter: off) for correction with next-generation sequencing data (4). For next-generation sequencing, MGIEasy Universal DNA Library Prep Kit V1.0 and MGIEasy DNA Clean beads were used following the standard protocol. Fragments 200–400 bp in size were end repaired and ligated adapters. High-quality clean reads of the DNBSEQ-T7RS platform were obtained using fastp (version: 0.12.6). The read length of PE150 was selected for error correction. In total, 1,250,938,800 bp clean reads were generated for CVCC519 and 1,179,368,400 bp for XM3104. After removing redundant reads, we used Circlator Version 1.5.5 (https://github.com/sanger-pathogens/circlator, parameter: fixstart) to move the origin of DnaA sequence to the start site of the genome replication to obtain the final genome (5).

Finally, one circular genome contig (4,749,670 bp and GC content 52.18%) and one circular plasmid (49,544 bp and GC content 52.10%) were found for CVCC519. One circular genome contig (4,749,611 bp and GC content 52.18%), one circular plasmid (49,544 bp and GC content 52.10%), and one potentially linear plasmid (118,995 bp and GC content 50.67%) were obtained for XM3104. Coding genes (coding DNA sequence [CDS]) were predicted using National Center for Biotechnology Information (NCBI) Prokaryotic Genome Annotation Pipeline (https://www.ncbi.nlm.nih.gov/genome/annotation_prok/) (6). A total of 4,593 and 4,701 CDSs were predicted for CVCC519 and XM3104 chromosome genomes, respectively. Drug resistance genes and virulence factors were annotated using the comprehensive antibiotic research database (CARD, https://card.mcmaster.ca/) (7) and the virulence factor database (VFDB, http://www.mgc.ac.cn/VFs/) (8). In all, 4,701 CDSs were detected for CVCC519 and 4,834 for XM3104. Thirty-nine antibiotic resistance genes and 131 virulence factors were found for both CVCC519 and XM3104.

Default parameters were used for all software except where otherwise specified.

## Data Availability

The complete genome sequences were deposited in National Center for Biotechnology Information (NCBI) under the accession numbers CP110934, CP110935, CP110931, CP110932, and CP110933. The raw sequences obtained from Nanopore PromethION sequencer and DNBSEQ-T7RS platform were deposited in the Sequence Read Archive (NGS of XM3104. NGS of CVCC519. PromethION sequence of XM3104. PromethION sequence of CVCC519.
